# Resveratrol, a New Allosteric Effector of Hemoglobin, Enhances Oxygen Supply Efficiency and Improves Adaption to Acute Severe Hypoxia

**DOI:** 10.3390/molecules28052050

**Published:** 2023-02-22

**Authors:** Zongtang Chu, Weidan Li, Guoxing You, Yuzhi Chen, Dong Qin, Peilin Shu, Yujing Wang, Ying Wang, Lian Zhao, Hong Zhou

**Affiliations:** Institute of Health Service and Transfusion Medicine, Academy of Military Medical Sciences, Academy of Military Science of the Chinese People’s Liberation Army, Beijing 100850, China

**Keywords:** hemoglobin oxygen affinity, P_50_ value, allosteric effect, Bohr effect, acute severe hypoxia, resveratrol

## Abstract

Acute altitude hypoxia represents the cause of multiple adverse consequences. Current treatments are limited by side effects. Recent studies have shown the protective effects of resveratrol (RSV), but the mechanism remains unknown. To address this, the effects of RSV on the structure and function of hemoglobin of adult (HbA) were preliminarily analyzed using surface plasmon resonance (SPR) and oxygen dissociation assays (ODA). Molecular docking was conducted to specifically analyze the binding regions between RSV and HbA. The thermal stability was characterized to further validate the authenticity and effect of binding. Changes in the oxygen supply efficiency of HbA and rat RBCs incubated with RSV were detected ex vivo. The effect of RSV on the anti-hypoxic capacity under acute hypoxic conditions in vivo was evaluated. We found that RSV binds to the heme region of HbA following a concentration gradient and affects the structural stability and rate of oxygen release of HbA. RSV enhances the oxygen supply efficiency of HbA and rat RBCs ex vivo. RSV prolongs the tolerance times of mice suffering from acute asphyxia. By enhancing the oxygen supply efficiency, it alleviates the detrimental effects of acute severe hypoxia. In conclusion, RSV binds to HbA and regulates its conformation, which enhances oxygen supply efficiency and improves adaption to acute severe hypoxia.

## 1. Introduction

**Figure 1:**
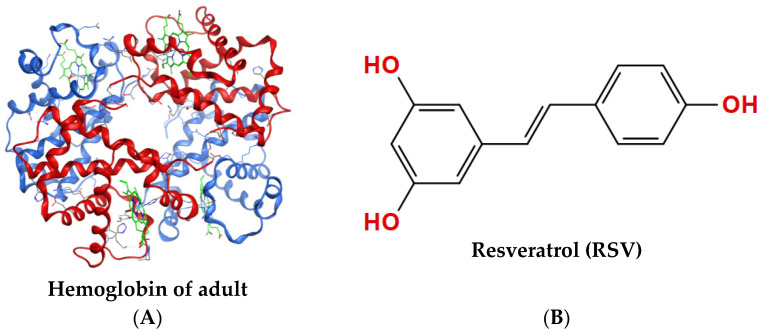
Structures of proteins and compounds. (**A**) Tetrameric structure of hemoglobin of adult (PDB 1GZX) with two α-chains shown in red, two β-chains shown in blue, and heme shown in green. (**B**) Molecular structure of resveratrol (RSV), CAS: 501-36-0.

Hemoglobin is a protein responsible for transporting oxygen in the blood of vertebrates. As shown in Figure 1A, the hemoglobin of adult (HbA) molecule comprises four subunits, each containing a globin chain and a heme group. Oxygen transport is the basic function of hemoglobin [1] and is mainly dependent on the capacity for hemoglobin binding and dissociation oxygen, referred to as hemoglobin–oxygen affinity [2,3]. The magnitude of hemoglobin–oxygen affinity is quantitatively characterized as the partial pressure of oxygen (PO_2_) at a hemoglobin oxygen saturation (SO_2_) rate of 50% (P_50_ value), which is one of the key parameters determining the oxygen supply efficiency of hemoglobin [4]. Increasing P_50_ values indicate that HbA is more likely to release oxygen, while decreasing values indicate a higher tendency for HbA to carry oxygen. Additional parameters representing the oxygen supply efficiency of HbA also include the Bohr effect (acid–base sensitivity index) and the theoretical oxygen-release capacity of HbA. The oxygen supply efficiency, as a key factor of HbA, is closely associated with adaption to oxygen-deficient environments, such as plateaus.

The atmospheric pressure and partial pressure of oxygen in plateau areas decrease with the increase in altitude. After suddenly entering a plateau area under emergency situations, there is a sharp decrease in the body’s oxygen intake which causes acute hypoxia and results in a series of complex pathological changes. Acute hypoxia can rapidly trigger headaches and dizziness and can even cause fatal pulmonary and cerebral edema, which makes emergency rescue missions in plateau areas challenging.

Hypoxic preconditioning and the use of Chinese traditional medicines in advance are steps commonly taken to alleviate acute hypoxia. At present, rhodiola is the main candidate used to prevent high-altitude hypoxia in China [5]. However, it is defined as a healthcare product, though the mechanism is unclear. Acetazolamide [6,7] and dexamethasone [8] are FDA-approved drugs used to prevent high-altitude hypoxia. Acetazolamide alleviates hypoxia symptoms by increasing bicarbonate excretion, stimulating the respiratory tract, and increasing the arterial partial pressure of oxygen [7]. Dexamethasone combats altitude sickness by reducing the blood volume in the brain and inhibiting lipid peroxidation [8]. However, an ideal drug to prevent or cure acute altitude hypoxia is urgently required.

Improving the capacity for oxygen uptake from the external environment and the oxygen-release capacity in peripheral tissues is considered a feasible strategy to prevent acute hypoxia damage; that is, the oxygen supply efficiency of hemoglobin should be improved [9]. Some drugs are capable of regulating the oxygen affinity of hemoglobin, such as GBT440 (trade-name Oxbryta) [10,11] and RSR-13 (trade-name Efaproxiral) [12,13,14,15]. As a drug used for sickle cell anemia, GBT440 can combine with sickle hemoglobin (HbS) to enhance oxygen affinity. However, because it does not improve blood stasis, it is difficult for oxygen to reach tissues, and thus oxygen transport to tissues decreases [16]. RSR-13 has the effect of reducing the oxygen affinity of hemoglobin, which improves the oxygen supply in local tissue. It is used as an adjunct therapy for the radiotherapy of patients with metastatic brain cancer [17]. RSR-13 may not be appropriate for preventing acute high-altitude hypoxia [18,19]. It is necessary to find an ideal drug to enhance the oxygen supply efficiency of hemoglobin at high altitudes.

Resveratrol (3,4′,5-trihydroxy-trans-stilbene, RSV) [20] is a nonflavonoid polyphenolic organic compound that is produced as an antitoxin by many plants when irritated, and it has the chemical formula C_14_H_12_O_3_, as shown in Figure 1B. RSV has antioxidant, anti-inflammatory, anticancer, and cardiovascular-protective effects, as shown by numerous ex vivo and in vivo experiments [21,22]. Recently, it has been studied for its effects in preventing acute altitude hypoxia. Deng et al. [23] established a rat model of the hypobaric-hypoxia-induced high-altitude polycythemia (HAPC). They found that RSV could effectively reduce the hemoglobin concentration of HAPC rats and improve the hemorheological index scores, enhance the antioxidant capacity, and improve the immune function of rats. The results suggested that RSV has an anti-hypoxic effect. Meanwhile, the binding of RSV to hemoglobin and the effect on its oxygenation have been mentioned in studies by Gualtieri et al. [24]. They suggested that the role of RSV in reducing erythrocyte results from its interaction with hemoglobin [23]. However, the effect of RSV on the oxygen supply efficiency of HbA remains unknown. The oxygen supply efficiency may be the drug’s target in relieving acute severe hypoxia, which requires further study.

In this study, we aimed to investigate the allosteric effect of RSV on HbA and verify the change in the oxygen supply efficiency. We then examined whether the anti-hypoxic effect of RSV is a result of improved oxygen supply efficiency. We expect to open up a new application for RSV by showing how it improves the oxygen supply efficiency in the field of high-altitude medicine.

## 2. Results

### 2.1. Resveratrol Binds to HbA with Concentration Gradients

**Figure 2:**
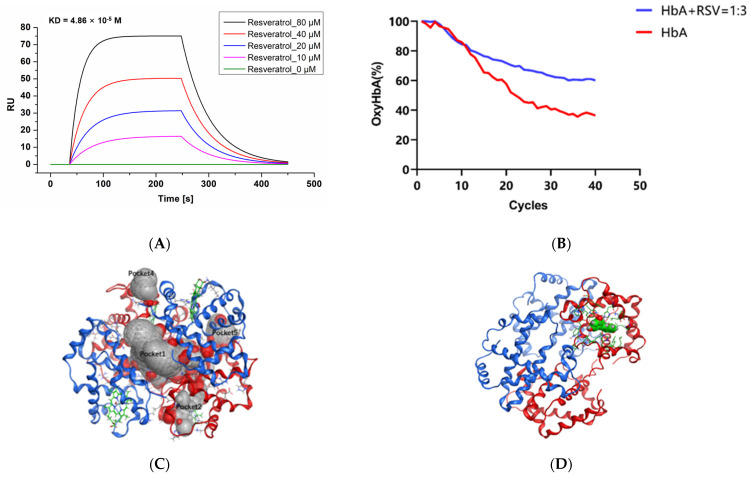

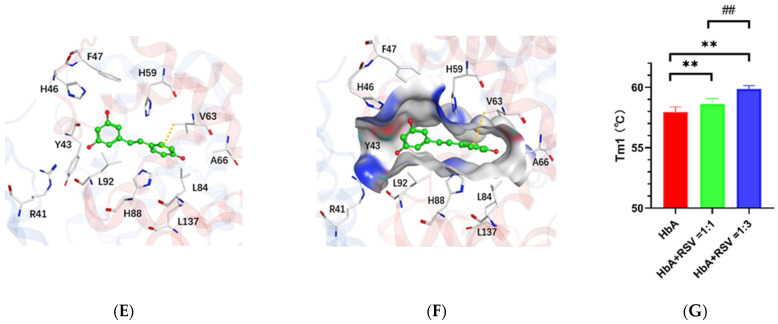
Ex vivo data on the interaction of RSV with HbA. (**A**) SPR assay results for RSV and HbA. The X-axis is time, and the Y-axis is resonance unit (RU). (**B**) Spectra of HbA with RSV over 4 h of deoxygenation. (**C**) Potential small-molecule binding pockets of the hemoglobin tetramer. In the protein structure, the hemoglobin α subunit homodimer is shown in red, the hemoglobin β subunit homodimer is shown in blue, and heme is shown in green. Small-molecule binding pockets were indicated by ellipses. (**D**) Hemoglobin/RSV complex. The tetramer is shown as a red and blue ribbon, and RSV is shown in green. (**E**) Resveratrol binding area and the contact amino acids (several amino acids are hidden for display convenience). (**F**) The molecular surface of the interaction region (red is the negatively charged region, blue is the positively charged region, and gray is the hydrophobic region). The amino acid V63 forms a Π-hydrogen bond with RSV. (**G**) Tm of HbA and HbA with RSV. Note: **, vs. HbA, *p* < 0.01; ##, vs. HbA: RSV = 1:1, *p* < 0.01.

The SPR assay results (Figure 2A) show that the resonance unit (RU) value increased as the RSV concentration was increased from 0 to 80 μM, which means that RSV combines with HbA according to a concentration gradient. By detecting changes in SPR angle, kinetic information such as the dissociation constant (KD) can be obtained, as shown in Table 1. KD (KD = Kd (dissociation constant)/Ka (association constant)) represents the dissociation degree of the ligand complex at equilibrium. The KD of RSV interacting with HbA was calculated as 4.86 × 10^−5^ M, which was higher than the KD value 4.62 × 10^−4^ M of 2,3-DPG, a classic endogenous allosteric effector, and HbA (Figure S1).

### 2.2. Resveratrol Delays the Oxygen Release of HbA

As shown in Figure 2B, we found that RSV delayed the transition of HbA from oxyHb to deoxyHb. During the first hour (Figure S3A), the rates of oxygen release from HbA and RSV were almost the same. However, from the second hour (Figure S3B) to the third hour (Figure S3C), the difference in the rates of oxygen release between HbA and HbA with RSV widened until the end (Figure S3D). After 4 h of N_2_ feeding, the oxyHb ratio of RSV-modified HbA decreased from 100% to 51.3 ± 4.89%, while in the control it decreased to 36.4 ± 6.24% (*p* < 0.05). RSV delayed the release of oxygen from HbA, which means it may cause reduced oxygen release in oxygen-rich environments, while increased oxygen reserves, in contrast, increase opportunities for release in hypoxic environments.

### 2.3. Resveratrol Binds the Heme Region of the Hemoglobin a Subunit

All docking poses were analyzed using the MOE package. Based on the docking regions, the potential small-molecule binding pockets on the surface of the tetramer were verified. Finally, the top five docking poses between the hemoglobin tetramer and RSV are shown in Figure 2C. Among them, pocket 1 is located at the interface of the tetramer. Pockets 2 and 3 are located in the heme binding region of the hemoglobin α subunit. Since they probe the binding pocket above the tetramer, pockets 2 and 3 are in the same region of two identical subunits. Pocket 4 is located at the interface of interaction between the hemoglobin ββ (C-terminal) and α subunits. Pocket 5 is located at the interface of interaction between the hemoglobin α (N-terminal) and β subunits. It can be seen in Figure S4A that RSV prefers to bind to pocket 2, known as the heme binding region. The optimal docked pose showed that the binding energy between RSV and hemoglobin is −5.76 kcal/mol. Further analysis of the optimal modes between RSV and the heme binding region is shown in Figure 2D,E. The hydrophobic RSV binding area and the phenol ring of RSV formed a Π-hydrogen bond with V63 amino acid of HbA. A group of hydrophobic or nonpolar amino acids and several positively charged amino acids, namely Y43, H46, and H59, were found to surround RSV, as shown in Figure 2F.

### 2.4. Resveratrol Stabilizes the Conformational Structure of HbA

As the temperature is increased, the structure of the HbA opens up gradually. In Figure S5A, the denaturation curve shows an obvious inflection point (Tm1) at 57.95 ± 0.74 °C for HbA. The addition of RSV at ratios of 1:1 and 1:3, respectively, induce shifting of the inflection point (Tm1) to 58.64 ± 0.75 ℃ (Figure S5B) (vs. HbA, *p* < 0.01) and 59.87 ± 0.46 °C (vs. HbA, *p* < 0.01; vs. addition of RSV at the ratio of 1:1, *p* < 0.01) (Figure S5C), which indicates that the oxyHbA conformation stabilizes following the addition of RSV (Figure 2G). 

### 2.5. Resveratrol Enhances the Oxygen Supply Efficiency Ex Vivo

**Figure 3:**
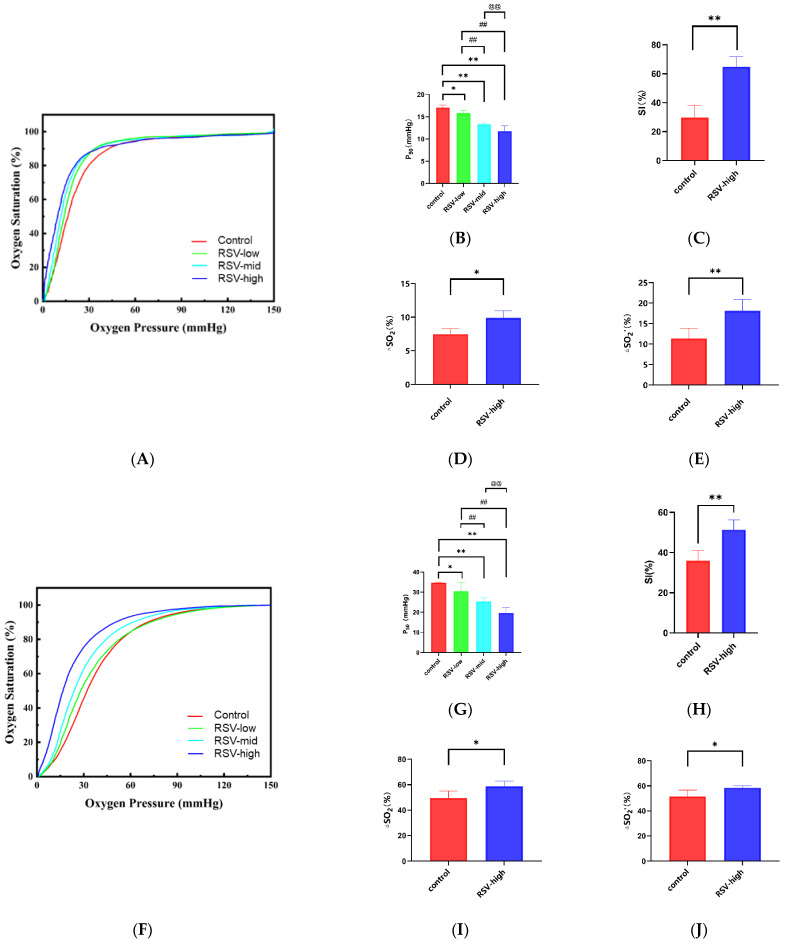
The oxygen supply efficiency of HbA and rat RBCs with RSV ex vivo. Oxygen dissociation curves of (**A**) HbA and (**F**) rat RBCs with different concentrations of RSV: P_50_ values of (**B**) HbA and (**G**) rat RBCs with different concentrations of RSV; SI values of (**C**) HbA and (**H**) and rat RBCs with different concentrations of RSV; ∆SO_2_ values after the modification of (**D**) HbA and (**I**) rat RBCs with different concentrations of RSV; ∆SO_2_′ values after the modification of (**E**) HbA and (**J**) rat RBCs with different concentrations of RSV. Note: *, vs. control, *p* < 0.05; **, vs. control, *p* < 0.01; ##, vs. RSV-low, *p* < 0.01; @@, vs. RSV-mid, *p* < 0.01.

As shown in Figure 3A,B, RSV decreases the P_50_ value of HbA. The P_50_ values of the HbA (control) and RSV-modified HbA (low-dose, mid-dose, and high-dose) groups were 19.48 ± 1.60 mmHg, 16.38 ± 1.38 mmHg (vs. control, *p* < 0.05), 15.64 ± 0.30 mmHg (vs. control, *p* < 0.01; vs. low-dose, *p* < 0.01), and 14.62 ± 1. 67 mmHg (vs. control, *p* < 0.01; vs. low-dose, *p* < 0.01; vs. mid-dose, *p* < 0.01), respectively. As shown in Figure 3F,G, the P_50_ value of the rat RBC (control) and RSV-modified RBC (low-dose, mid-dose, and high-dose) groups were 34.71 ± 0.41 mmHg, 30.56 ± 4.23 mmHg (vs. control, *p* < 0.05), 25.37 ± 1.84 mmHg (vs. control, *p* < 0.01; vs. low-dose, *p* < 0.01), and 19.60 ± 2.68 mmHg (vs. control, *p* < 0.01; vs. low-dose, *p* < 0.01; vs. mid-dose, *p* < 0.01), respectively. It follows that the effect of the high-dose group is the most significant. As shown in Figure 3C, the SI value was significantly higher for the RSV-modified HbA group (high-dose) than for the HbA group (64.93 ± 6.89% vs. 29.77 ± 8.42%, *p* < 0.01). Similarly, as shown in Figure 3H, the SI value for the RSV-modified rat RBCs was 51.36 ± 4.91%, which was significantly higher than for the rat RBCs (35.93 ± 4.93%, *p* < 0.01). As shown in Figure 3D,E, the theoretical oxygen-release capacity under plain conditions (∆SO_2_) for the RSV-modified HbA (high-dose) was significantly higher than for HbA (9.89 ± 1.02% vs. 7.47 ± 0.80%, *p* < 0.05). The theoretical oxygen-release capacity under plateau conditions (∆SO_2_′) for the RSV-modified HbA (high-dose) was significantly higher than for HbA (18.14 ± 2.79% vs. 11.34 ± 2.55%, *p* < 0.01). Similarly, as shown in Figure 3I,J, the theoretical oxygen-release capacity under plain conditions (∆SO_2_) for RSV-modified rat RBCs (high-dose) was 58.70 ± 4.24%, which was significantly higher than for rat RBCs (49.49 ± 5.40%, *p* < 0.05). The theoretical oxygen-release capacity under plateau conditions (∆SO_2_′) for the RSV-modified rat RBCs (high-dose) was 58.42 ± 1.60%, which was significantly higher than for rat RBCs (51.52 ± 5.11%, *p* < 0.05). Resveratrol thus significantly enhances oxygen affinity, the Bohr effect, and theoretical oxygen-release capacity values of HbA and RBCs ex vivo.

### 2.6. Resveratrol Significantly Increases The Survival Time of Mice under Acute Hypoxic Asphyxia

**Figure 4:**
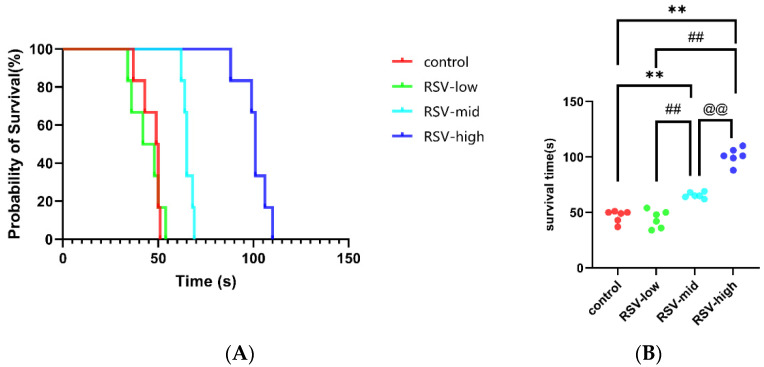
The survival times of mice injected with RSV under acute hypoxic asphyxia: (**A**) probability of survival in acute hypoxic asphyxia; and (**B**) changes in survival times of mice injected with different concentrations of RSV under acute hypoxic asphyxia. Note: **, vs. control, *p* < 0.01; ##, vs. RSV-low, *p* < 0.01; @@, vs. RSV-mid, *p* < 0.01.

As shown in Figure 4A, after 7 days of injections, RSV (mid-dose and high-dose) can significantly increase the survival time of mice under acute hypoxic asphyxia. The details are as shown in Figure 4B. The survival time of mice under acute anaerobic conditions changed from 46.67 ± 5.54 s to 44.00 ± 8.00 s for the low-dose group (vs. control, *p* > 0.05), 65.50 ± 2.59 s for the mid-dose group (vs. control, *p* < 0.01; vs. low-dose, *p* < 0.01), and 100.80 ± 7.47 s for the high-dose group (vs. control, *p* < 0.01; vs. low-dose *p* < 0.01; vs. mid-dose *p* < 0.01), indicating that RSV can improve the ability for tolerating acute hypoxic asphyxia.

### 2.7. Resveratrol Enhances Adaption to Acute Severe Hypoxia

#### 2.7.1. Resveratrol Inhibits Acute Hypoxia-Induced Weight Loss

**Figure 5:**
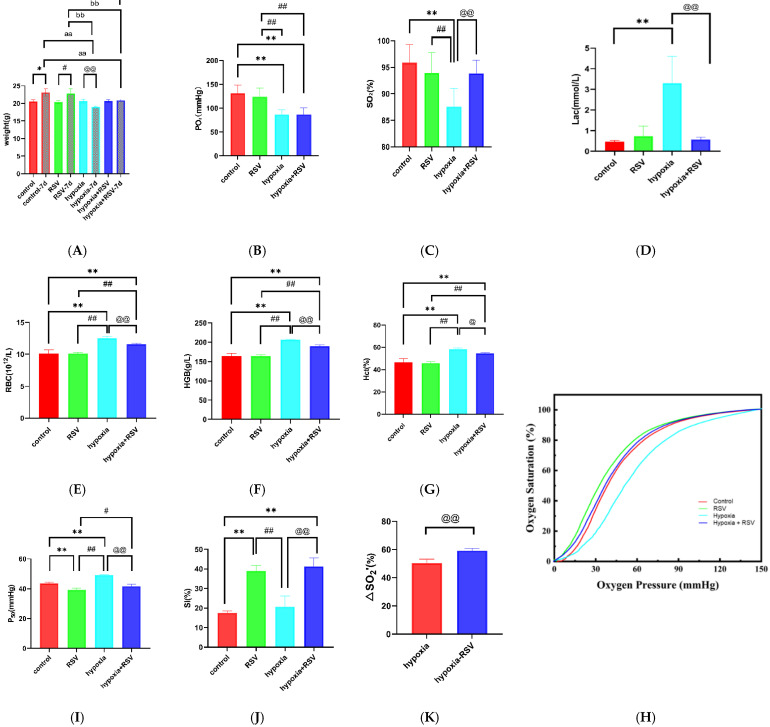
Hypoxic adaption of mice treated with RSV: changes in body weight (**A**), arterial blood gas analysis results for the partial pressure of oxygen (PO_2_) (**B**), arterial oxygen saturation (SO_2_) results (**C**), lactate counts (**D**), blood cell analysis results for counts of red blood cells (RBCs) (**E**), hemoglobin volume (HGB) values (**F**), counts of hemoglobin (Hct) (**G**), oxygen dissociation curves (**H**), P_50_ values (**I**), SI (**J**), and ∆SO_2_′ values (**K**) after 7 days in the control group, normoxia with RSV group, hypoxia group, and hypoxia with RSV group. Note: *, vs. control group, *p* < 0.05; **, vs. control group, *p* < 0.01; #, vs. normoxia with RSV group, *p* < 0.05; ##, vs. normoxia with RSV group, *p* < 0.01; @, vs. hypoxia group, *p* < 0.05; @@, vs. hypoxia group, *p* < 0.01; aa, vs. control-7d group, *p* < 0.01; bb, vs. normoxia with RSV group, *p* < 0.01; cc, vs. hypoxia group, *p* < 0.01.

As shown in Figure 5A, after 7 days, the control group BALB/c mice gained weight from 20.60 ± 0.40 g to 23.14 ± 1.02 g (*p* < 0.05). The normoxia with RSV mice also showed a change in weight, from 20.36 ± 0.47 g to 22.84 ± 1.29 g (*p* < 0.05). The weight of the mice decreased from 20.66 ± 0.56 g to 19.04 ± 0.23 g in the hypoxia group (*p* < 0.01). The weight was basically stable from 20.72 ± 0.34 g to 20.88 ± 0.26 g in the hypoxia with RSV group (*p* > 0.05). The body weight increased in both groups under normoxia, while it decreased in mice under hypoxia—an effect that was abolished when they were treated with RSV.

#### 2.7.2. Resveratrol Increased the Arterial Oxygen Saturation and Inhibited the Lactate Increase under Acute Hypoxia

As shown in Figure 5B, the PO_2_ value for the control group was 131.3 ± 17.61 mmHg, while for the normoxia with RSV group it was 123.3 ± 18.44 mmHg (vs. control group, *p* > 0.05). The PO_2_ value for hypoxia group mice decreased to 86.58 ± 10.19 mmHg (vs. control group and normoxia with RSV group, *p* < 0.01), while the PO_2_ value for the hypoxia with RSV group was 87.02 ± 13.40 mmHg (vs. control group and normoxia with RSV group, *p* < 0.01; vs. hypoxia group, *p* > 0.05). As shown in Figure 5C, the SO_2_ value for the control group was 95.88 ± 3.43%, while for the normoxia with RSV group it was 93.97 ± 3.84% (vs. control group, *p* > 0.05). For the hypoxic mice, the value was 87.53 ± 3.47% (vs. control group and normoxia with RSV group, *p* < 0.01), while the administration of RSV under hypoxia increased the SO_2_ to 93.88 ± 2.50% (vs. control group and normoxia with RSV group, *p* > 0.05; vs. hypoxia group, *p* < 0.01). Through these observations, the decreases in PO_2_ and SO_2_ in the hypoxia group indicate the development of hypoxia. As shown in Figure 5D, the cLac value of the control group was 0.47 ± 0.06 mmol/L, while the cLac value of the normoxia with RSV group was 0.73 ± 0.49 mmol/L (vs. control group, *p* > 0.05). The cLac value for the hypoxia group mice increased to 3.30 ± 1.31 mmol/L (vs. control group, *p* < 0.01), while that of the hypoxia with RSV group decreased to 0.57 ± 0.12 mmol/L (vs. hypoxia group, *p* < 0.01).

#### 2.7.3. Resveratrol Inhibits the Increases in RBCs and Hemoglobin during Acute Severe Hypoxia

The RBC count for the mice in the control group was 10.14 ± 0.54 × 10^12^, while the RSV injection resulted in a slight decrease to 10.08 ± 0.25 × 10^12^ (*p* > 0.05). The RBC count for the mice in the hypoxia group increased to 12.54 ± 0.30 × 10^12^ (vs. control group and normoxia with RSV group, *p* < 0.01). After the administration of RSV, the RBC count decreased to 11.62 ± 0.13 × 10^12^ (vs. control group, normoxia with RSV group and hypoxia group, *p* < 0.01). The HGB value for the mice in control group was 164.0 ± 7.35 g/L, while it increased to 164.6 ± 3.72 g/L due to RSV injection, although the change was not statistically significant (*p* > 0.05). The HGB value for the hypoxic mice increased to 206.5 ± 1.29 g/L (vs. control group and normoxia with RSV group, *p* < 0.01). After the administration of RSV under hypoxia, the HGB value decreased to 190.3 ± 3.40 g/L (vs. control group, normoxia with RSV group, and hypoxia group, *p* < 0.01). The Hct value for the control mice was 46.78 ± 2.83%, while it decreased to 45.88 ± 1.48% (vs. control group, *p* > 0.05) following RSV injection. The Hct value for the hypoxic mice increased to 58.18 ± 1.15% (vs. control group and normoxia with RSV group, *p* < 0.01). After the administration of RSV under hypoxia, the Hct value decreased to 54.63 ± 0.62% (vs. control group, *p* < 0.01, and normoxia with RSV group, *p* < 0.01; vs. hypoxia group, *p* < 0.05); that is, under acute severe hypoxia, the RBC count, Hct, and HGB values were significantly increased, which were restored by RSV through the anti-hypoxic effect.

#### 2.7.4. Resveratrol Enhances the Oxygen Supply Efficiency In Vivo

As shown in Figure 5H, the oxygen dissociation curve (ODC) showed a shift to the left after RSV administration compared with the control group. We also found that after acute severe hypoxia, the ODC was shifted to the right compared with the control group. Interestingly, after RSV treatment in the hypoxia group, the ODC shifted to the left compared with the hypoxia group and was similar to the control group. As shown in Figure 5I, the P_50_ value for the control mice was 43.61 ± 0.73 mmHg, while it decreased to 39.19 ± 1.18 mmHg (vs. control group, *p* < 0.01) following RSV treatment. Hypoxia gave a P_50_ value of 49.08 ± 0.28 mmHg (vs. control group and normoxia with RSV group, *p* < 0.01), while it decreased to 41.74 ± 1.36 mmHg (vs. control group, *p* > 0.05, vs. normoxia with RSV group, *p* < 0.05; vs. hypoxia group, *p* < 0.01) following RSV treatment. As shown in Figure 5J, the SI value for the control mice was 17.44 ± 1.06%, while it increased to 38.89 ± 2.95% (vs. control group, *p* < 0.01) in response to RSV, indicating that RSV enhances the Bohr effect in treated mice. The SI value for the hypoxia mice was 20.62 ± 5.56% (vs. control group, *p* > 0.05; vs. normoxia with RSV group, *p* < 0.01), while RSV increased it to 41.29 ± 4.26% (vs. control group, *p* < 0.01; vs. normoxia with RSV group, *p* > 0.05; vs. hypoxia group, *p* < 0.01). As shown in Figure 5K, the theoretical oxygen-release capacity under plateau conditions (∆SO_2_′) for hypoxia mice was 50.40 ± 2.77%, while RSV increased it to 59.22 ± 1.62% (*p* < 0.01); that is, RSV enhances oxygen affinity and the Bohr effect in mice under normoxia and acute hypoxia, which is in line with our expectations. Moreover, RSV enhances the oxygen supply efficiency of mice in an acute severe hypoxic state.

#### 2.7.5. Resveratrol Protected the Organ Damage Caused by Acute Hypoxia

**Figure 6:**
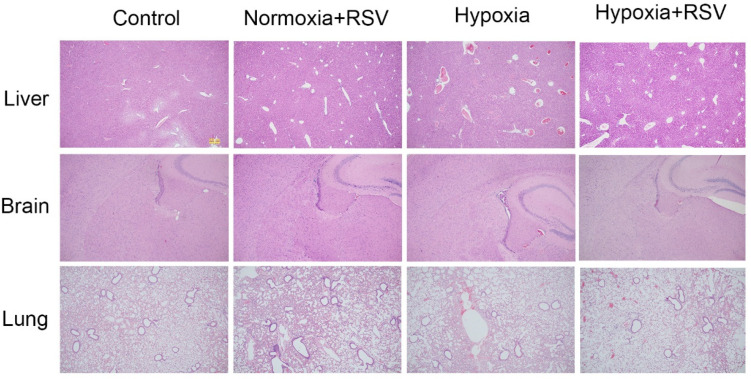
H&E analysis of liver, brain, and lung tissues of mice in the normal, normal with RSV, hypoxia, and hypoxia with RSV groups.

As shown in Figure 6, the adaption of acute hypoxia via RSV intervention was determined using immunohistochemistry analysis of the lung, brain, and liver tissues of the mice. Compared with the normal group, an increased number of dilated portal and central veins with stasis were induced by hypoxia, while RSV intervention significantly reduced the number of portal veins and central veins. Although dilated and congested central veins could also be observed in the RSV-treated hypoxia group, no obvious cases of focal necrosis or swollen hepatocytes were detected. Hypoxia induced the disorganization of the pyramidal neuron cell layer in the hippocampal region of the brain tissue, reduced the number of pyramidal cells, and caused vascular expansion with RBC extravasation, while after the RSV intervention, the layers of pyramidal neurons in the hippocampal region were more neatly arranged, with consistent cell volumes and an increased number of pyramidal cells. For the hypoxia group, alveolar wall thickening and the fusion of alveolar cavities into large alveoli were detected. Moreover, the expansion of small interstitial vessels and widening of the alveolar septum were also induced by hypoxia. After RSV intervention, there was an alveolar structure in lung tissue, the lumen expansion of interstitial small vessels was more limited, RBCs were rarely in the lumen, and the alveolar septum was less widened.

## 3. Discussion

### 3.1. Resveratrol Is an Allosteric Effector of HbA and Enhances the Oxygen Supply Efficiency of Blood Ex Vivo

Resveratrol has been well known as a protector agent against oxidative protein damage [25,26,27,28]. However, in recent years, the correlation between RSV and HbA has attracted increasing attention. Galtieri et al. found that RSV can interact with hemoglobin, prompting a transition in T–R conformation toward the higher-affinity R state [24]. Through molecular docking, the putative binding site for RSV was found, located at the central cavity of hemoglobin of bovine, and the calculations indicate greater binding specificity, which correlates well with the effect of RSV in the functional modulation of HbA [26,29]. It was also shown that RSV can cross the membranes of RBCs and bind to HbA, with alterations to the ATP release and metabolism of these RBCs [26]. Meanwhile, Deng’s work showed that RSV suppresses the development of polycythemia resulting from hypoxia [23]. Hemoglobin in RBCs plays a vital role in oxygen transfer. Thus, all of these results pushed us to clarify the effects of RSV on the oxygen supply efficiency of hemoglobin and the adaption of acute hypoxia.

On the basis of the existing research, we experimentally verified the physical association between HbA and RSV. According to the SPR assay, RSV has a concentration gradient binding effect with HbA, which makes it possible that RSV has an allosteric effect. Yu Lin Jiang [30] mentioned the binding values of RSV and Human Serum Albumin (HSA), Ka = 1.64 ± 0.07 × 105 M^−1^, KD = 1/Ka = 6.1 μM. The objects and detection methods in that study were not consistent with our work. The KD value for RSV binding to hemoglobin is not mentioned in these previous studies or in any other studies, and we are reporting it for the first time. Similar methods were used in 38,700 molecular protein interactions, but only TD-1 [31] has been mentioned as a hemoglobin allosteric effector. Therefore, the effect of RSV on HbA in the delivery of oxygen was confirmed by the ODA test, an emerging type of high-throughput assay [32,33]. The data show a significant reduction in the rate of oxygen release for HbA after RSV treatment, which preliminarily indicates a change in HbA function. Furthermore, molecular docking was carried out. Hemoglobin itself is an ~64.5 kDa globular protein consisting of two α-chains and two β-chains, which each form complexes with one heme molecule, representing the key site for oxygen binding [34]. The data showed that RSV is able to bind to multiple regions of HbA while it acts mainly on the pocket of heme according to the binding energy, which may be the mechanism through which RSV promotes oxygen supply efficiency. The pocket of heme and the positively charged amino acids Y43, H46, and H59 are related to the binding of hydrogen ions, which indirectly explains the action of RSV in enhancing the Bohr effect [35,36]. We believe that the phenol ring of RSV forms a Π-hydrogen bond with V63, which mainly results in the enhanced binding ability of oxygen with heme. It is worth noting that the results of a previous docking study [29] are somewhat different from our results in showing that RSV is located in the proximity of the central cavity of the hemoglobin. The difference may have resulted from the different docking methods. Here, we applied an induced fit algorithm to study the binding of RSV to the four small-molecule binding pockets of HbA. This approach actually enriches the molecular docking data and, thus, allows for a more solid conclusion. However, experimental assays such as mutagenesis testing should be further performed to confirm the binding mode. Moreover, with the increase in Tm1, it is believed that the structure of HbA becomes more stable by binding with RSV, which also shows a dose-dependent effect. 

The key point of the present paper is the effect of RSV on the oxygen supply efficiency of HbA and rat RBCs, which was systematically analyzed for the first time. Previous studies have found that RSV has an effect on hemoglobin–oxygen affinity [29]. Based on this, we not only used an ODA test but also carried out a systematic oxygen binding and dissociation analysis method for verification. Our data show that RSV significantly reduces the P_50_ values of both the HbA and rat RBCs; that is, it enhances hemoglobin–oxygen affinity and increases the retention of oxygen in oxygen-deficient tissue. Additionally, our data show that the SI values increase as the Bohr effect was enhanced, which also means that RSV enhances oxygen release in oxygen-deficient tissue. Most critically, the theoretical oxygen-release capacity of HbA was also increased via binding with RSV under simulated plateau conditions, suggesting that hemoglobin delivers more oxygen to the body after RSV treatment. The results indicate that RSV enhances oxygen supply efficiency and is a candidate for generating anti-hypoxic effects under plateau conditions.

Allosteric effectors often show different modulatory effects because they play different roles in oxygen supply. Hemoglobin–oxygen affinity changes following binding to ligand molecules. The 2,3-DPG compound is a classic endogenous allosteric effector that reduces hemoglobin–oxygen affinity. A variety of new exogenous hemoglobin effectors are constantly being developed. For example, RSR-13 [37] and ITPP [38] can cause decreased oxygen affinity, while TD-3 [39] and 5-HMF [40] can increase oxygen affinity. However, they all stalled in preclinical or clinical studies because of inadequate regulatory effect. Based on the ability for oxygen supply efficiency regulation, many studies have focused on the adaption to acute hypoxia. Dominelli et al. [41] proposed that individuals with high oxygen affinity are better adapted to hypoxia at high altitudes, while many other studies have proposed that the enhanced Bohr effect is beneficial to acute hypoxia at high altitudes [42,43]. Many molecules such as GBT440 [44], GBT1118 [45], and 5-HMF [46] have also appeared in hypoxia studies, and their effect of reducing P_50_ values is thought to be correlated with enhanced hypoxia resistance. Therefore, an allosteric effector may be used to enhance oxygen affinity and the Bohr effect of HbA as well as improve the theoretical oxygen-release capacity under plateau (hypoxic) conditions, thus facilitating adaption to acute altitude hypoxia. In the present study, RSV showed surprising effects in terms of enhancing oxygen affinity and the Bohr effect and increasing the theoretical oxygen-release capacity in a simulated plateau environment with HbA and rat RBCs ex vivo. The results suggest that RSV may be an effective anti-hypoxic drug under plateau conditions. 

### 3.2. Resveratrol Improves Adaption to Acute Severe Hypoxia

To further verify the effects of RSV against hypoxia, the effects of RSV on acute asphyxia tolerance and acute hypoxia adaption in mice were analyzed. We found that RSV prolongs the survival time of mice with extreme hypoxic asphyxia and enhances the ability to carry oxygen under hypoxic conditions, which are consistent with the ex vivo results. We examined the oxygen supply efficiency of hypoxic mice and analyzed the changes after the RSV treatment. Increased P_50_ values after acute hypoxia have been mentioned in the literature [47]. We found that RSV could prevent increased P_50_ values while increasing the SI. Furthermore, RSV significantly enhanced the theoretical oxygen-release capacity values in vivo. All changes resulted in the prolongation of the period of acute asphyxia tolerated by mice under anaerobic conditions. These results confirm the relevance of RSV in regulating HbA function and improving adaption to acute hypoxia.

Further simulating the severe hypoxic environment, we also observed that the anti-hypoxic ability of mice significantly increases after the RSV injection. The concentration of intraperitoneal injection was summarized from previous studies [48,49,50,51]; different studies have used up to 10–100 mg/kg. We simulated a severe hypoxic environment with an oxygen content range of 9.5–10.5%, which is close to the plateau environment at an altitude of 6000 m [52,53]. In our results, RSV had positive effects on the mice suffering from the hypoxic environment in terms of allowing them to sustain body weight and maintain stable lactate concentrations. The anti-hypoxic effect of RSV is further supported by the SO_2_ values, pathological analysis results, hemoglobin concentrations, and RBC count changes. SO_2_ is one of the key indicators reflecting oxygen supply and consumption in the body. RSV significantly increased SO_2_ in hypoxic mice, which may have been due to the effect of RSV on the oxygen supply efficiency. The pathological damage to the organs is an important evaluation index of hypoxia. In this study, we found that RSV plays a protective role in the brain, liver, and lungs, especially in terms of attenuating the pathological angiogenesis and hemorrhage caused by hypoxia. Increased RBC counts and hemoglobin concentrations are the classic symptoms of hypoxia. In agreement with the reports by Deng et al. [23], we also confirmed the inhibitory effect of RSV on hemoglobin after hypoxia. Furthermore, we measured the red blood cell and hematocrit counts, and the results are consistent with the hemoglobin concentration.

### 3.3. Enhancement of the Oxygen Supply Efficiency of Blood Improves Adaption to Acute Hypoxia

After suffering from acute hypoxia, natural compensation increases RBCs count (hemoglobin) to supply oxygen to tissues, which causes irreversible damage to cardiopulmonary function [54]. Therefore, we need to find a direct and effective way to regulate the blood oxygen supply. Direct adjustment of hemoglobin not only improves the oxygen supply but also avoids the side effects of increased RBCs, which makes the use of allosteric effectors an ideal strategy for treating acute hypoxia. 

This study clarified that the structure of the heme region in hemoglobin changes specifically after the binding of RSV, which enhances the oxygen supply efficiency of HbA, increasing oxygen affinity, the Bohr effect, and theoretical oxygen-release capacity. The results further indicate that enhancing oxygen affinity has special effects in extreme hypoxic environments [55]. This is consistent with the trend in the evolutionary direction for the Tibetan population and some high-altitude organisms [47]. Meanwhile, enhancement of the Bohr effect can increase the ability of the body to obtain oxygen under extreme anoxic environments [42]. So far, only a few allosteric effectors, such as 2,3-DPG [56] and ITPP [57], have been identified, indicating that there are few candidates to increase the Bohr effect. The increased theoretical oxygen-release capacity can reflect the enhanced oxygen release of hemoglobin [1]. Therefore, the results indicate that RSV enhances oxygen supply efficiency and improves adaption to acute severe hypoxia by binding to HbA.

## 4. Methods and Materials

### 4.1. Ethical Considerations

All experimental procedures were approved by the Laboratory Animal Center of the Academy of Military Medical Sciences (IACUC-DWZX-2022–631, Beijing, China). The research protocol adhered to the institutional guidelines for the care and use of laboratory animals.

### 4.2. Sample Preparation

#### 4.2.1. Compounds

Resveratrol (CAS:501-36-0) was purchased from Targetmol (Burlington, MA, USA). For all studies herein, the compounds were solubilized in 100% dimethyl sulfoxide (DMSO; Sigma Aldrich, St. Louis, MO, USA) at a concentration range of 9 μM–100 mM.

#### 4.2.2. Hemoglobin of Adult and Rat Red Blood Cells

Human whole-blood samples were withdrawn from the median cubital veins of volunteers who were six healthy men, 28–36 years old. Then, 50 mL of blood was mixed with citrate phosphate dextrose adenine (CPDA-1; Sigma Aldrich, St. Louis, MO, USA), and the final concentration of CPDA-1 was 14%. Hemoglobin was purified via anion-exchange chromatography, as previously described [58].

Six healthy male Wistar rats (220–260 g; Vital River, Beijing, China) with ad libitum access to food and water were anesthetized by an intraperitoneal injection of 50 mg/kg of sodium pentobarbital sodium (Chinese Medicine Group Chemical Agent, Beijing, China) and placed in the supine position on a warming pad (TMS-202, Softron Biotechnology, Beijing, China), with the temperature maintained at 37 ± 0.1 °C. Heparin (400 U/kg; Chinese Medicine Group Chemical Agent, Beijing, China) was administered via the carotid artery to inhibit coagulation. Blood collection, storage, and leukoreduction were performed as described in our previous work [58]. 

### 4.3. Surface Plasmon Resonance (SPR) Assays

Surface plasmon resonance (SPR) assays were performed using an OpenSPR instrument (Nicoya Life Science, Inc., Kitchener, ON, Canada). The COOH sensor chip (Nicoya SEN-AU-100-12-COOH, Kitchener, ON, Canada) was prepared prior to the experiment. HEPES running buffer (with 1% DMSO, pH 7.4), immobilization buffer (10 mM sodium acetate, pH 4.5), 400 mM of 1-(3-dimethylaminopropyl)-3-ethylcarbodiimide hydrochloride (EDC), 100 mM of N-hydroxysuccinimide (NHS), and blocking buffer (1 M ethanolamine) were prepared using reagents obtained from Sigma Aldrich, St. Louis, MO, USA. We mixed 400 mM of EDC with 100 mM of NHS immediately after preparation of solutions to activate the COOH chip and then diluted HbA in immobilization buffer (6 mg/mL). Then, the HbA solution was injected at a flow rate of 20 μL/min for 420 s. The chip was blocked using 1 M ethanolamine hydrochloride at a flow rate of 20 μL/min for 240 s. Resveratrol was diluted in running buffer to 10 µM and injected into the flow cell of the channel at a flow rate of 20 μL/min for an association period of 240 s, followed by 240 s for dissociation. The association and dissociation processes were handled in the running buffer. The analysis software used in this experiment was TraceDrawer (Ridgeview Instruments lab, Uppsala, Sweden). The data were analyzed using the one-to-one analysis model [59]. The 2,3-Diphosphoglycerate (2,3-DPG) compound was used as control. The detailed method is shown in the supporting information.

### 4.4. Oxygen Dissociation Assay (ODA) 

According to Patel [33], ODA is a novel screening assay based on the spectral changes observed during HbA deoxygenation, and oxyHbA% is calculated according to Formula (1) [60]. Purified HbA (3 µM of tetramer in various buffers) was incubated for 1 h under ambient air at 37 °C in the presence or absence of RSV (molar ratio of 1:3) in 96-well optically transparent polystyrene plates (SARSTEDT, Inc., Nümbrecht, Germany). After incubation, the samples were deoxygenated with gaseous dry N_2_ for 4 h at 37 °C in an ultraviolet/visible (UV/Vis) absorbance spectrometer (Omega, BMG Labtech, Inc., Ortenberg, Germany) capable of reading full spectra within 1 s/well. Deoxygenation was achieved by blowing N_2_ over (but not directly into) the samples at 20 L/min. During the process of buffer and HbA equilibration during deoxygenation, spectral measurements (350–700 nm, with a spectral resolution of 1 nm) were obtained every 6 min as a cycle to determine the oxyHb level over time [33].

(1) $$\mathrm{oxyHbA}\%=\frac{1.013\times \left(A576-A700\right)-0.3269\times \left(A630-A700\right)-0.7353\times \left(A560-A700\right)}{0.478\times \left(A576-A700\right)+1.9211\times \left(A630-A700\right)+0.478\times \left(A560-A700\right)}$$

### 4.5. Molecular Docking

The crystal structure 1GZX (https://www.rcsb.org/structure/1GZX (accessed on 20 May 2022)), at the resolution of 2.1 Å, represents human hemoglobin HBA/HBB with oxygen in the form of a tetramer in the T State. It was selected for analyzing the interaction between HbA and RSV. First, 1GZX was protonated and optimized using the MOE plug-in “quickprep” [61]. The potential small-molecule binding pockets on the surface of 1GZX were detected using the MOE plug-in ‘SiteFinder’, and the quality of the pockets was evaluated based on the propensity of ligand binding (PLB) score. Among them, five pockets with PLB scores higher than 0.1 were selected for molecular docking. The 3D structures and multiple conformations of RSV were generated using the ‘conformational search’ program in the MOE software (version 2020.9).

The induced fit docking protocol was used, in which the triangle match algorithm was used to generate the docking mode, the London δG was used as the scoring function to calculate the binding energy for each docking pose, and the top 30 docking poses were retained. Finally, these docking poses were further optimized under the induced fit algorithm using the GBVI/WAS δG scoring function. The binding affinity for the optimized docking poses was then calculated [62,63,64,65]. The 2,3-DPG compound has been proven able to bind to HbA forming the HbA–2,3-DPG complex based on X-ray resolution (the crystal structure 1B86, https://www.rcsb.org/structure/1B86 (accessed on 11 February 2022)), which could be utilized as control. The detailed method is shown in the supporting information.

### 4.6. Thermal Stability Experiments

The stability of HbA before and after binding to RSV was evaluated using the UNcle All-in-One Biologics Stability Screening Platform (Unchained Labs, Norton, MA, USA). Purified HbA was diluted to 1 mg/mL with PBS. Then, 9 mL of HbA (with RSV at molar ratios of 1:1 and 1:3) solution was loaded into the sample well to measure the melting midpoint temperature (Tm1). The HbA solution was heated from 25 to 95 °C at a rate of 0.25 °C/min, and the fluorescence was detected every 20 s. The instrument generates the barycentric mean (BCM) of the intrinsic fluorescence spectra versus the temperature. The first-order derivative of the BCM curve was calculated and plotted against the temperature to generate the derivative curve; the temperature corresponding to the first peak of the derivative curve represents Tm1. The Tm1 and BCM of the maximum emission wavelength of fluorescence (Emax) were calculated using the UNcle Analysis software (Unchained Labs, Norton, MA, USA, V.4.0) [66,67].

### 4.7. Oxygen Supply Efficiency Tests Ex Vivo

Here, 3 mg of HbA or rat RBCs with the same amount of hemoglobin were co-incubated with RSV at molar ratios of 1:1, 1:3, and 1:6 for low-dose, mid-dose, and high-dose concentrations, respectively. An oxygen binding–dissociation analyzer (BLOODOX-2018, Softron, Beijing, China) was used to record hemoglobin–oxygen dissociation curves and obtain the P_50_ value [58]. At the same time, we obtained measurements for determining the acid–base sensitivity index (SI) and the theoretical oxygen-release capacity. All three parameters represent the oxygen supply efficiency of HbA or rat RBCs. The SI was calculated according to Formula (2) [68]. P_50_ acid is the P_50_ of ODC in acidic conditions (pH = 7.2); P_50_ base is the P_50_ of ODC in alkaline conditions (pH = 7.6); and P_50_ neutral is the P_50_ of ODC at pH = 7.4. The theoretical oxygen-release capacity is defined as the difference in oxygen saturation under different pH and PO_2_ concentrations, calculated based on the oxygen dissociation curve according to Formula (3) or (4). The ex vivo calculation method for oxygen release based on the oxygen dissociation curve fully considers the influence of the different environments. 

SI:

(2) $$\mathrm{SI}=\left(P_{50}\;\mathrm{acid}-P_{50}\;\mathrm{base}\right)/P_{50}\;\mathrm{neutral}\;\times 100\%$$

Theoretical oxygen-release capacity in plain conditions:

(3) $$\Delta {\mathrm{SO}}_{2}={\mathrm{SO}}_{2}\;\left(\mathrm{pH}=7.6,{\;\mathrm{PO}}_{2}=100\;\mathrm{mmHg}\right)-{\mathrm{SO}}_{2}\;\left(\mathrm{pH}=7.2,{\;\mathrm{PO}}_{2}=40\;\mathrm{mmHg}\right)$$

Theoretical oxygen-release capacity in plateau conditions:

(4) $${∆{\mathrm{SO}}_{2}}^{\prime }={\mathrm{SO}}_{2}\;\left(\mathrm{pH}=7.6,{\;\mathrm{PO}}_{2}=60\;\mathrm{mmHg}\right)-{\mathrm{SO}}_{2}\;\left(\mathrm{pH}=7.2,{\;\mathrm{PO}}_{2}=30\;\mathrm{mmHg}\right)$$

Here, ∆SO_2_ represents the theoretical oxygen-release capacity of HbA in plain environments and ΔSO_2_′ represents the theoretical oxygen-release capacity of HbA in plateau environments. SO_2_ (pH = 7.6, PO_2_ = 100 mmHg) represents the HbA oxygen saturation at pH = 7.6 and a partial pressure of oxygen at 100 mmHg, which simulates the lungs under plain conditions. SO_2_ (pH = 7.2, PO_2_ = 40 mmHg) represents the HbA oxygen saturation at pH = 7.2 and a partial pressure of oxygen at 40 mmHg, which simulates the periapical tissue under plain conditions. SO_2_ (pH = 7.6, PO_2_ = 60 mmHg) represents the HbA oxygen saturation at pH = 7.6 and a partial pressure of oxygen at 60 mmHg, which simulates the lungs under plateau conditions (altitude 6000 m). SO_2_ (pH= 7.2, PO_2_ = 30 mmHg) represents the HbA oxygen saturation at pH = 7.2 and a partial pressure of oxygen at 30 mmHg, which simulates the periapical tissue under plateau conditions.

### 4.8. Acute Hypoxic Asphyxia Tolerance Detection

Twenty-four healthy male BALB/c mice (20.0–20.6 g; Vital River, Beijing, China) with ad libitum access to food and water were randomly divided into four groups. One group was injected with 2% DMSO and the others were intraperitoneally injected with RSV (10 mg/kg, 50 mg/kg, or 100 mg/kg with 2% DMSO) once a day for 7 days. On the 7th day, the mice were anesthetized via an intraperitoneal injection of 2.5% pentobarbital sodium at 50 mg/kg and placed in a small closed chamber (280 mm × 180 mm × 210 mm). We created an absolutely oxygen-free environment by feeding the chamber with a steady flow (10 L/min) of N_2_ consecutively at room temperature. The changes in heart rate for the mice were observed through the tail using an intelligent non-invasive sphygmomanometer (Softron, Beijing, China). When the heart rate remained at 0 for 10 consecutive seconds, the mice were defined as having succumbed to asphyxia death, and the time at which this occurred was recorded.

### 4.9. Anti-Acute Hypoxia Detection

Twenty-four healthy male BALB/c mice (20.0–20.6 g; Vital River, Beijing, China) with ad libitum access to food and water were randomly divided into four groups: (1) control; (2) normoxia with RSV; (3) hypoxia; (4) hypoxia with RSV. The hypoxia groups were fed in the hypoxic chamber (Aipu, Hangzhou, China), which was set to 25 ℃ for 7 days. Nitrogen gas was fed at a flow rate of 1 L/min to gradually maintain the oxygen content range at 9.5–10.5%. This was done to simulate the environment at 6000 m above sea level, forming an acute severe hypoxia state. For the RSV group, RSV was injected intraperitoneally at a concentration of 100 mg/kg once daily. The weight was recorded every day. Seven days later, the arterial blood gas was detected using a blood gas analyzer (ABL90 FLEX, Radiometer, Brønshøj, Denmark) and a blood cell analysis was performed using a hematology analyzer (Mindray, Shenzhen, China) immediately after arterial blood was drawn from the carotid artery of each mouse. The changes in PO_2_, SO_2_, cLac, RBC count, hematocrit (Hct), and hemoglobin (HGB) values were analyzed and the oxygen supply efficiency indicators (P_50_, SI, theoretical oxygen-release capacity) were observed. Then, the mice were sacrificed and subjected to H&E pathological analysis to detect changes in the liver, brain, and lungs before and after hypoxia.

### 4.10. Statistical Analysis

Data are shown as means ± standard deviations (SDs). Statistical analyses between groups were performed using one-way analysis of variance (ANOVA) or Kruskal–Wallis one-way analysis when heterogeneity of variance was observed. Unpaired Student’s *t*-test was used for the assessment of statistically significant differences between the two groups in terms of ODA, SI, and theoretical oxygen-release capacity values. Survival data were analyzed using the log-rank test. Statistical analysis was performed using GraphPad (GraphPad Prism version 9, GraphPad Software, La Jolla, CA, USA), with *p* < 0.05 considered to indicate statistically significant differences.

## 5. Conclusions

We verified the interaction between the HbA and RSV concentration gradients and found that RSV enhances the oxygen supply efficiency of HbA ex vivo and in vivo, which facilitates adaption to acute severe hypoxia.

## Figures and Tables

**Table 1:** Ka, Kd, and KD values of HbA and RSV calculated using SPR assays.

Parameter	Value
Ka (association constant)	3.97 × 10^2^
Kd (dissociation constant)	1.93 × 10^−2^
KD (dissociation equilibrium constant)	4.86 × 10^−5^ M

## Data Availability

Not applicable.

## Supplementary Materials

The following supporting information can be downloaded at: https://www.mdpi.com/article/10.3390/molecules28052050/s1, Figure S1: SPR assay results for 2,3-DPG and HbA; Figure S2: the 3D structures of 2,3-DPG in the docked pose and the crystal structure (PDBID: 1B86). The RMSD value and the docking score were indicated; Figure S3: Spectra of HbA with RSV over 4 h of deoxygenation; Figure S4: Molecular docking of RSV and HbA; Figure S5: Thermal stability of HbA with RSV; Table S1: Ka, Kd, and KD values of HbA and 2,3-DPG calculated using SPR assays.
