# Supplementary material for: Resveratrol, a New Allosteric Effector of Hemoglobin, Enhances Oxygen Supply Efficiency and Improves Adaption to Acute Severe Hypoxia
Source: Molecules. 2023 Feb 22;28(5):2050. doi: 10.3390/molecules28052050 (PMC10004267; doi:10.3390/molecules28052050)
Supplement: Supplementary file 1 [file molecules-28-02050-s001.zip › molecules-2199443-supplementary.pdf]

molecules MDPI

Supplementary Materials

Resveratrol, a New Allosteric Effector of Hemoglobin, Enhances Oxygen Supply Efficiency and Improves Adaption to Acute Severe Hypoxia

Zongtang Chu, Weidan Li, Guoxing You, Yuzhi Chen, Dong Qin, Peilin Shu, Yujing Wang, Ying Wang *, Lian Zhao * and Hong Zhou *

Institute of Health Service and Transfusion Medicine, Academy of Military Medical Sciences, Academy of Military Science of the Chinese People's Liberation Army, Beijing 100850, China

* Correspondence: wy830111@163.com (Y.W.); zhaolian@bmi.ac.cn (L.Z.); zhouhtt1966@163.com (H.Z.)

Supplementary methods and results

- Surface plasmon resonance (SPR) assays

Surface plasmon resonance (SPR) assays were performed using an OpenSPR instrument (Nicoya Life Science, Inc., Kitchener, Canada). The COOH sensor chip (Nicoya SEN-AU-100-12-COOH, Kitchener, Canada) was prepared prior to the experiment. HEPES running buffer (with 1% DMSO, pH7.4), immobilization buffer (10 mM sodium acetate, pH4.5), 400 mM of 1-(3-dimethylaminopropyl)-3-ethylcarbodiimide hydrochloride (EDC), 100 mM of N-hydroxysuccinimide (NHS), and blocking buffer (1 M ethanolamine) were prepared using reagents obtained from Sigma Aldrich, St Louis, MO, USA. We mixed 400 mM of EDC with 100 mM of NHS immediately after preparation of solutions to activate the COOH chip, then diluted HbA in immobilization buffer (6 mg/mL). Then, the HbA solution was injected at a flow rate of 20 μL/min for 420 s. The chip was blocked using 1 M ethanolamine hydrochloride at a flow rate of 20 μL/min for 240 s. 2,3-DPG was diluted in running buffer to 400 μM and injected into the flow cell of the channel at a flow rate of 20 μL/min for an association period of 240 s, followed by 240 s for dissociation. The association and dissociation processes were handled in the running buffer. The analysis software used in this experiment was TraceDrawer (Ridgeview Instruments lab, Sweden). The data were analyzed using the one-to-one analysis model [26]. As shown in figureS2, HbA captured on COOH chip can bind 2,3-DPG with an affinity constant of 462 μM as determined in a SPR assay.

Table S1. Ka, Kd, and KD values of HbA and 2,3-DPG calculated using SPR assays.

Parameter	Value
Ka (association constant)	$2.32e^{1}$
Kd (dissociation constant)	$1.07e^{-2}$
KD (dissociation equilibrium constant)	$4.62e^{-4}$ M

- Molecular docking of 2,3-DPG to hemoglobin based on 1B86

Mauguen, Y. et al proved that 2,3-diphosphoglycerate (2,3-DPG) was able to bind to hemoglobin and formed the hemoglobin-2,3-diphosphoglycerate complex, which was solved by x-ray (1). In order to test the feasibility of the molecular docking procedure, compound 2,3-DPG was docked to the experimental determined area of hemoglobin by using the flexible docking protocol as described in methods and materials of molecular docking. Top five docked poses were reserved and the docking scores were shown in Figure S2, where RMSD value is the root mean square deviation of the compound 2,3-DPG between the docked pose and the crystal structure (1B86). The final docking score was calculated as S value, where the smaller of S, the stronger of the binding affinity.

Based on the molecular docking results, the RMSD value of the compound 2,3-DPG between the five docked poses and the crystal structure (1B86) were 4.84, 2.25, 2.89, 2.77, 6.32 Å respectively. The differences between RMSD values may be caused by water solvents, which were not included in the docking process. The docking scores were -6.21, -6.20, -5.99, -5.80, -5.77 kcal/mol in each docked pose (Figure S2).

Supporting Figures

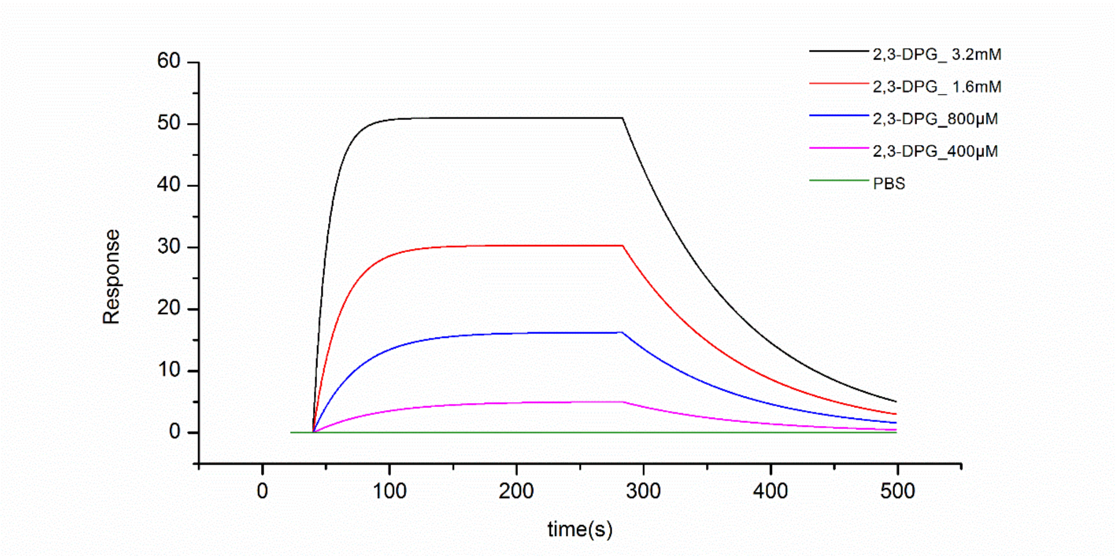

Figure S1. SPR assay results for 2,3-DPG and HbA.

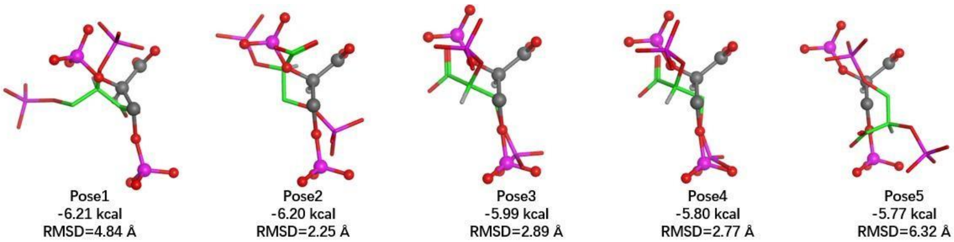

Figure S2. the 3D structures of 2,3-DPG in the docked pose and the crystal structure (PDBID: 1B86). The RMSD value and the docking score were indicated.

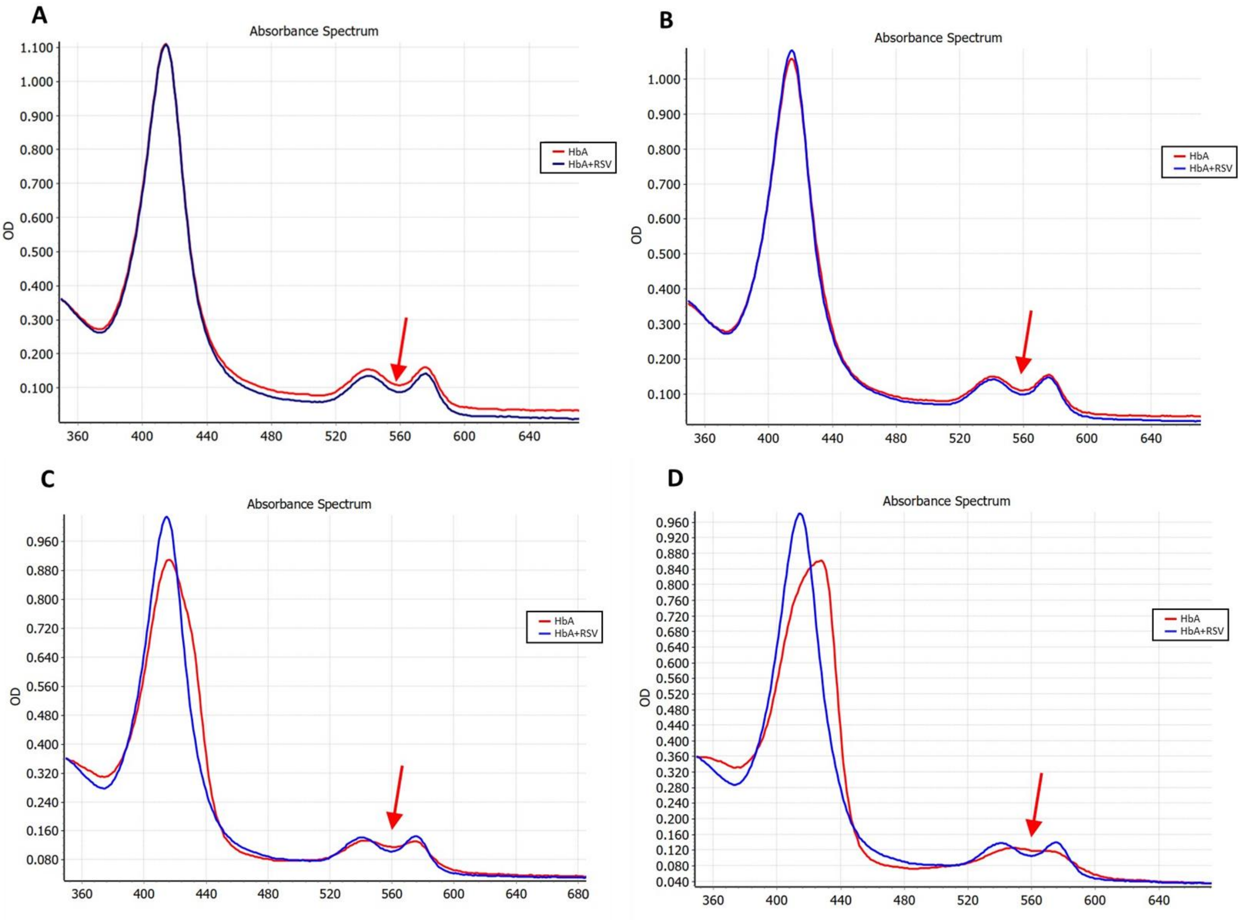

Figure S3. Spectra of HbA with RSV over 4 hours of deoxygenation: (A) spectra of HbA (red) and HbA+RSV (blue) over 1 hour (10 cycles) of deoxygenation; (B) spectra of HbA (red) and HbA+RSV (blue) over 2 hours (20 cycles) of deoxygenation; (C) spectra of HbA (red) and HbA+RSV (Blue) over 3 hours (30 cycles) of deoxygenation; (D) spectra of HbA (red) and HbA+RSV (blue) over 4 hours (40 cycles) of deoxygenation. The red arrows point out the change in the characteristic spectrum of oxyhemoglobin during deoxygenation.

A

ID	S_Pocket1	rmsd_refine	E_conf	E_place
T1558	-5.2630	1.5373	-47.3707	-91.5190
T1558	-5.2546	1.0884	-45.8026	-65.2198
T1558	-5.2343	1.0882	-48.0935	-92.0875
T1558	-5.2200	0.6344	-47.7644	-69.6105
T1558	-5.2136	1.2590	-47.8836	-75.8711

ID	S_Pocket2	rmsd_refine	E_conf	E_place
T1558	-5.7627	2.9184	-47.2835	-71.5108
T1558	-5.7054	2.1906	-47.7960	-85.1973
T1558	-5.6988	1.8258	-47.6660	-73.8478
T1558	-5.5880	2.5138	-48.4269	-76.3169
T1558	-5.5402	1.6619	-48.2070	-75.2394

ID	S_Pocket4	rmsd_refine	E_conf	E_place
T1558	-4.2840	2.6355	-48.0968	-3.0991
T1558	-4.2442	0.9812	-47.8697	-36.5306
T1558	-4.2220	0.8092	-47.9091	-37.4789
T1558	-4.2041	1.5001	-47.9004	-56.6413
T1558	-4.1663	1.9533	-47.9237	-14.3252

ID	S_Pocket5	rmsd_refine	E_conf	E_place
T1558	-5.1608	1.3138	-48.2694	-53.5949
T1558	-5.1114	3.3775	-48.4255	-62.0982
T1558	-4.8829	2.6733	-47.5806	-55.6395
T1558	-4.8228	0.7877	-46.0770	-83.7420
T1558	-4.7301	1.0396	-47.4379	-56.2935

B

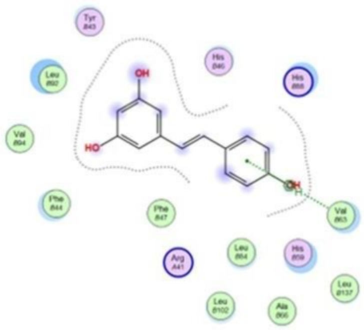

Figure S4. Molecular docking of RSV and HbA. (A) Docking scores of RSV (T1558) to the HBA/HBB tetramer; rmsd_refine: the root means square deviation between the pose before refinement and the pose after refinement (Å); E_ Conf: the energy of the conformer. If there is a refinement stage, this is the energy calculated at the end of the refinement. E_ Place: the conformational energy during molecular docking. The final docking score is calculated as the S value, where the smaller the S, the stronger the binding affinity. (B) The 2D binding mode of the hemoglobin/RSV complex, with a docking score of -5.76 kcal/mol.

A

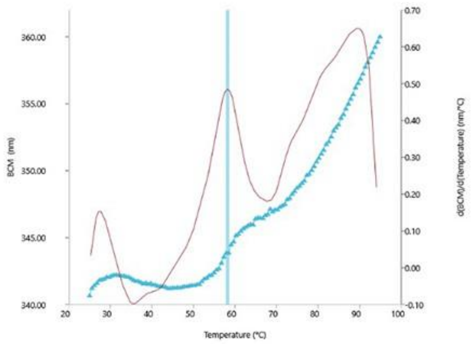

B

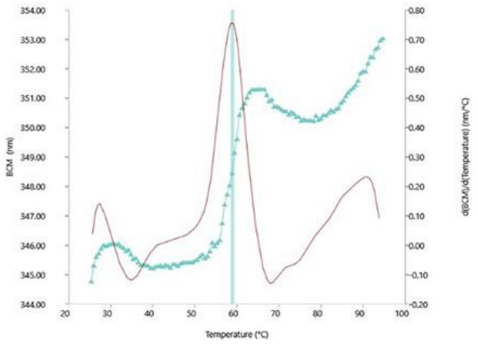

C

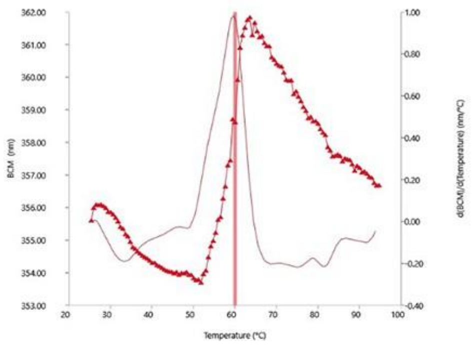

Figure S5. Thermal stability of HbA with RSV: (A) denaturation curves of HbA; (B) denaturation curves of HbA: RSV=1:1; (C) denaturation curves of HbA vs. RSV=1:3.
